# Correction: Inhibition of PKCβ2 overexpression ameliorates myocardial ischaemia/reperfusion injury in diabetic rats via restoring caveolin-3/Akt signaling

**DOI:** 10.1042/CS-20140789_COR

**Published:** 2021-12-08

**Keywords:** calveolin-3, diabetes, myocardial ischaemia/reperfusion injury

The authors would like to address an incorrect labelling in Figure 3C in the original publication of this paper in *Clinical Science* (2015) **129**(4), 331–344; DOI: https://doi.org/10.1042/CS20140789. The ischemia reperfusion model was ‘ischemia for 30 minutes and reperfusion for 120 minutes’. However Figure 3C in the original publication was incorrectly labelled as ‘reperfusion for 30 minutes’. Therefore, the labelling for Figure 3C should be the same as in Figure 3D to reflect that R(min) is 120.

